# Whole-genome regulation analysis of histone H3 lysin 27 trimethylation in subclinical mastitis cows infected by *Staphylococcus aureus*

**DOI:** 10.1186/s12864-016-2947-0

**Published:** 2016-08-08

**Authors:** Yanghua He, Minyan Song, Yi Zhang, Xizhi Li, Jiuzhou Song, Yuan Zhang, Ying Yu

**Affiliations:** 1 Key Laboratory of Animal Genetics, Breeding and Reproduction, Ministry of Agriculture, National Engineering Laboratory for Animal Breeding, College of Animal Science and Technology, China Agricultural University, Beijing, 100193 People’s Republic of China; 2 Department of Animal & Avian Sciences, University of Maryland, College Park, MD 20742 USA; 3 Beijing Sanyuan Breeding Technology Co. Ltd., Capital Agribusiness Group, Beijing, China

**Keywords:** Dairy cattle, H3K27me3 regulation, Subclinical mastitis, *Staphylococcus aureus*

## Abstract

**Background:**

*S. aureus* is one of the major etiological agents causing bovine subclinical mastitis. The regulatory effects of H3K27me3 on gene expression in subclinical *S. aureus* mastitis cows are unknown. This study aimed to profile genome-wide transcriptional changes regulated by H3K27me3 in bovine lymphocytes applied in subclinical *S. aureus* mastitis cows and healthy controls.

**Results:**

A total of 61 differentially expressed genes (DEGs) were detected in subclinical *S. aureus* mastitis cows compared to the healthy controls, of which 25 DEGs are up-regulated and the rest are down-regulated genes in subclinical *S.aureus* mastitis cows. The up-regulated genes are mainly involved in the Jak-STAT signaling pathway, cytokine-cytokine receptor interaction, and T cell receptor-signaling pathway, while the down-regulated genes are related to metabolism pathways. Combination analysis of histone methylation and gene expression revealed that H3K27 trimethylation levels in silent genes were higher in subclinical *S. aureus* mastitis cattle than in healthy cows. The key regions of H3K27me3 target genes related to subclinical *S. aureus* mastitis were the upstream 2 kb regions of the DEGs relative to transcription start site (TSS).

**Conclusions:**

The current study provides a novel insight into the interaction between *S. aureus* and lymphocytes in lactating cows by histone H3 methylation regulation. The differentially expressed genes in bovine lymphocytes regulated by H3K27me3 on upstream 2 kb regions (*IL10, PTX3* and etc.) may relate to *S. aureus* mastitis susceptibility and could be considered as key candidate genes for anti- *S. aureus* mastitis study and breeding.

**Electronic supplementary material:**

The online version of this article (doi:10.1186/s12864-016-2947-0) contains supplementary material, which is available to authorized users.

## Background

Subclinical mastitis is one of the major challenging diseases to the modern dairy industry [1]. It affects both quantity and quality of milk [2–5]. *S. aureus* mastitis is an extremely complex disease in cattle due to immune defenses of the host, antibiotic resistance, hidden attacks and the propensity of recurrence [6, 7]. Indeed, *S. aureus* mastitis not only affects dairy cattle but also forms a serious threat to public health because the organisms that cannot be destroyed by heat treatment would potentially cause food poisoning by producing enterotoxins in the milk [8, 9, 10].

Improvements of management and udder health in few developed countries seem to have reduced prevalence of *S. aureus* mastitis [11]. However, the widespread prevalence of *S. aureus* in the environment suggests that it is an unlikely the pathogen to be eradicated [12, 13]. Therefore, deciphering the interaction between cows and *S. aureus* infection would make controlling this complex disease in dairy cattle become more practical.

Trimethylation of lysine 27 on histone H3 (H3K27me3) is the major part of epigenetic modifications which bridges hosts across pathogens [14]. H3K27me3 as a transcription-suppressor is normally related with silencing of gene expression. In mammals, H3K27me3 is catalyzed by proteins of the polycomb group (PcG), which are an evolutionally conserved set of long-term transcriptional gene repressors [15, 16]. Moreover, H3K27me3 was found to play a vital role in development, imprinting, carcinogenesis, and inflammatory diseases [17].

The modification of H3K27me3 in bovine was found mostly in embryonic development [18–20]. In 2012, our group reported the genome-wide H3K27me3 modification profiles in bovine lymphocytes [21]. However, the regulatory function of H3K27me3 in *S. aureus* mastitis in dairy cattle has not been determined yet [22]. Keeping in view the importance of H3K27me3, the present study was conducted to find out the genome-wide regulatory effects of H3K27me3 on gene expression in *S. aureus* subclinical mastitis cows and healthy cows, and to analyze the functions of H3K27me3 modification in *S. aureus* subclinical mastitis susceptibility and resistance in dairy cattle.

## Results

### The identification of subclinical mastitis dairy cattle caused by *S. aureus*

**Fig. 1:**
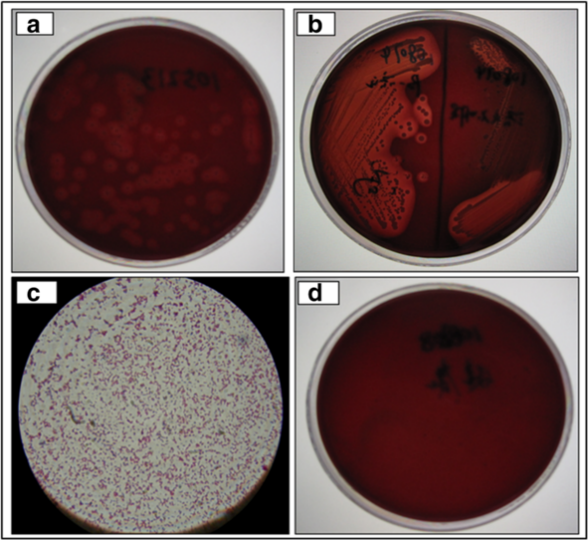
*S. aureus* culture from cattle milk samples. **a** Positive results of *S. aureus* culture of milk samples on a plate of blood agar. **b** Purified culture of *S. aureus.* **c** Gram stains for *S. aureus.* **d** Negative results of *S. aureus* culture of milk samples on a plate of blood agar

**Fig. 2:**
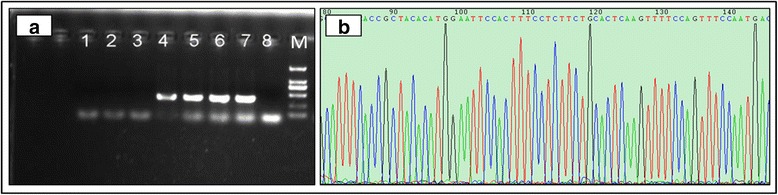
Identification of *S. aureus* in milk samples. **a** PCR amplification of *nuc* gene. Lanes 1, 2, 3 and 8 were *S. aureus*-negative; lanes 4, 5, 6 and 7 were *S. aureus*-positive; M: Marker I. **b** 16S rRNA gene sequencing of genomic DNA of *S. aureus*

There are invisible inflammatory changes in subclinical cows’ udders, except for a drop in milk production [23]. Considering *S. aureus* infection is the main reason to cause subclinical mastitis [24], the object of the present study was subclinical mastitis Holstein cows induced by *S. aureus* based on a series of bacteria identification. We selected seventeen Holstein cows as candidate subclinical mastitis dairy cattle based on their dairy herd improvement (DHI) records throughout the whole lactation period. The fresh milk samples were collected aseptically from these cows for somatic cell count (SCC) and bacteria identification. The result of bacteria culture showed as suspected that *S. aureus* (Fig. 1b) and gram stain was positive (Fig. 1c) for mastitis cattle while the number of bacteria detected was quite small for healthy cows (Fig. 1d). We also used molecular detection to identify subclinical *S. aureus* mastitis. The sequence of the *nuc* gene that encodes the thermostable nuclease of *S. aureus* was amplified by the polymerase chain reaction (PCR) to detect *S. aureus* mastitis [25]. For milk samples infected by *S. aureus*, a band with the length of 279 bp was observed on agarose gel electrophoresis, and there were no any bands for samples uninfected by *S. aureus* (Fig. 2a). For further confirmation, bacterial sequence was detected by 16S rRNA gene sequencing method using universal primers (Additional file 1: Table S1) and the specific sequence of *S. aureus* was identified by NCBI-blast (Fig. 2b). Finally, we were relatively confident in confirming the milk samples infected by *S. aureus*. Of seventeen candidate mastitis cattle, seven cows were healthy without any bacteria detected (41 %), three cows were infected by *S. aureus* (18 %), three cows were infected by *staphylococcus haemolyticus* (18 %), two cows were infected by *streptococcus agalactiae* (12 %), one was infected by *Serratia marcescens* (6 %) and the other one was infected by mixed bacteria (6 %). Three mastitis cows were infected by *S. aureus* and three healthy cows without any bacteria were chosen as our experimental cattle. Peripheral blood was collected, and high throughput sequencing of the lymphocytes was conducted for these six cows (see Methods).

### The gene expression profiles of subclinical *S. aureus* mastitis and healthy dairy cattle

High throughput sequencing was performed for the six cows to obtain gene expression levels of all annotated bovine genes. Around 3.5 million clean sequence tags were generated, and the number of unambiguous tag-mapped genes was more than 6000 for these six individuals (Additional file 1: Table S2). Our experimental individuals were divided into two groups: *S. aureus* mastitis group (SS1, SS3 and SS4 individuals) and healthy group (SH2, SH5 and SH6), to identify the differentially expressed genes related to *S. aureus* mastitis infection.

**Fig. 3:**
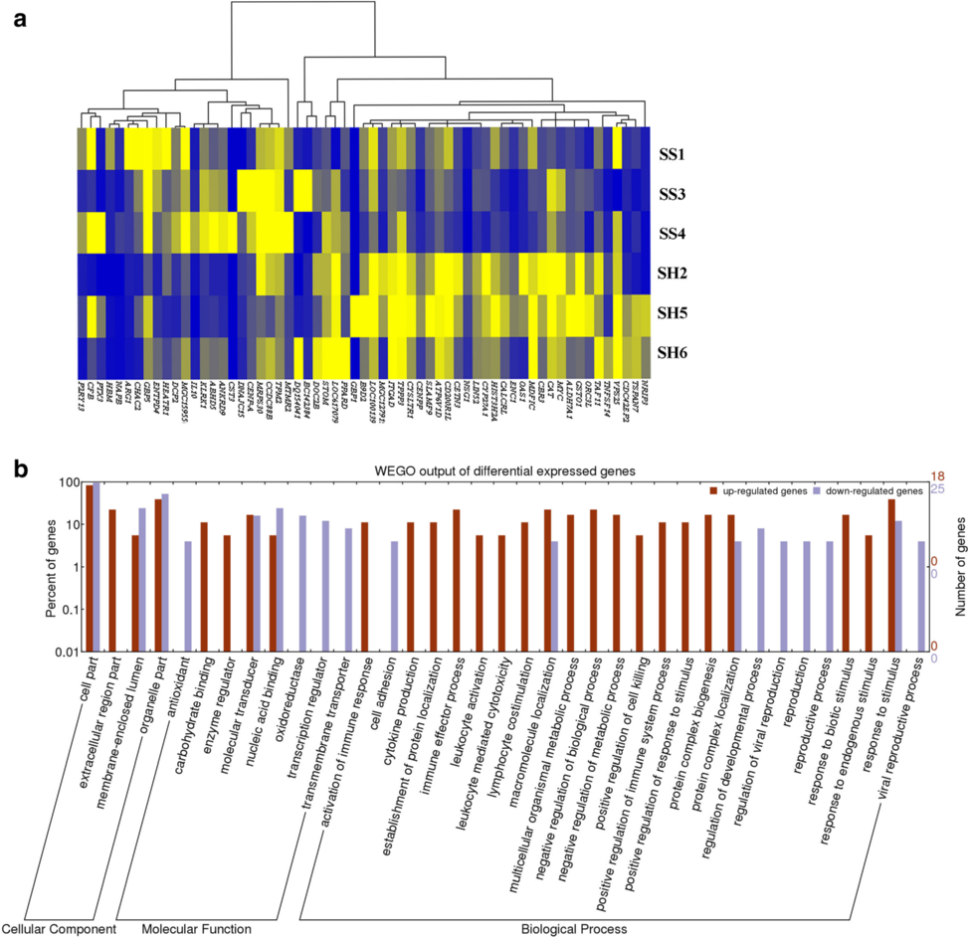
The differentially expressed genes associated with susceptibility of subclinical *S. aureus* mastitis. **a** Cluster analysis of differentially expressed genes between *S. aureus* mastitis and healthy cows. From gene expression data, 25 up-regulated and 36 down-regulated genes were identified between *S. aureus* mastitis cows (SS1, SS3 and SS4) and healthy cows (SH2, SH5 and SH6). Normalized intensity values of genes (columns) were ordered using Centroid Spearman Rank Correlation and hierarchical clustering in Cluster3.0 software. The dendrogram showed the similarity (distance) of mRNA expression levels and was divided into sub-trees as distinguished from different colors. Arrays (rows) were grouped by six different individuals. Yellow and blue colors reflect the high and low expression intensities, respectively. **b** GO analysis of differentially expressed genes. Genes were grouped by cell component, molecular function and biological process based on the bovine GO annotation information. Gene numbers and percentages (on log scale) are listed for each category

A total of 61 differentially expressed genes was identified based on the criteria of FDR ≤ 0.05 and |log2Ratio| ≥ 1, in which 25 were up-regulated genes and the other 36 were down-regulated genes. Cluster analysis of differentially expressed genes (Fig. 3a) and GO analysis were conducted in DAVID database. The results indicated that these up- and down-regulated genes have specific GO enriched terms, in which up-regulated genes that were highly expressed in mastitis cows compared to healthy dairy cattle are mainly involved in immune-related processes, such as *KLRK1* gene which is associated with activating signaling pathways of cell surface receptors by the innate immune response. The *KLRK1* gene also participates in lymphocyte co-stimulation and NK cell mediated cytotoxicity. *CFB* is related to complement activation. *IL10* is involved in secretion of cytokine and synthesis of interferon in immune responses. *PTX3* was observed in the immune effect process and response to fungus. *ARG1* was correlated with host response to bacterial source molecular. However, down-regulated genes were mainly involved in the oxidation-reduction enzyme activities, transmembrane transporter activities and transcription regulator activities, as well as regulation of developmental and reproductive processes (Fig. 3b).

To explore the signaling pathways that these differentially expressed genes are involved in, KEGG pathway analysis of the differentially expressed genes was conducted. Our results showed that the up-regulated genes are associated with the Jak-STAT signaling pathway, cytokine-cytokine receptor interaction, natural killer cell mediated cytotoxicity and T cell receptor signaling pathway (Additional file 1: Table S3). The down-regulated genes mainly participate in metabolism-related and disease-related pathways. Therefore, these differentially expressed genes associated with immune responses and diseases might play vital roles in mastitis susceptibility, suggesting that they could be candidate genes for mastitis prevention.

### Genome-wide identification of H3K27me3 regions in subclinical *S. aureus* mastitis and healthy dairy cattle

**Fig. 4:**
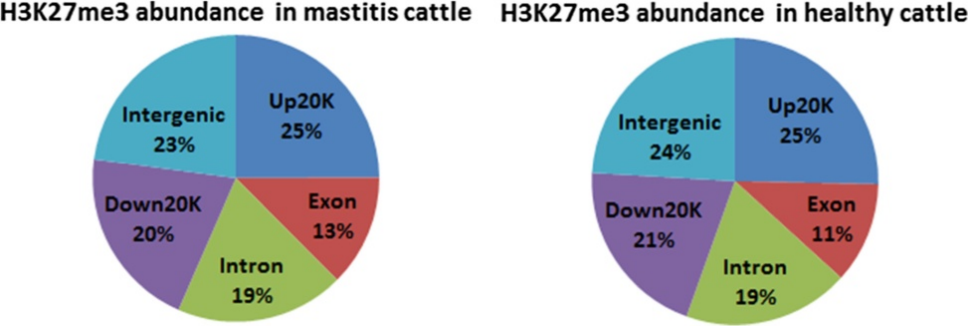
The percentages of the abundance of bovine lymphocytes H3K27me3 reads among five genomic regions. The bovine genome was divided into five kinds of regions for each gene: 20 kb upstream of transcription start site (TSS), exon (including 5′and 3′UTR), intron, 20 kb downstream of transcription end site (TES) and intergenic regions

**Fig. 5:**
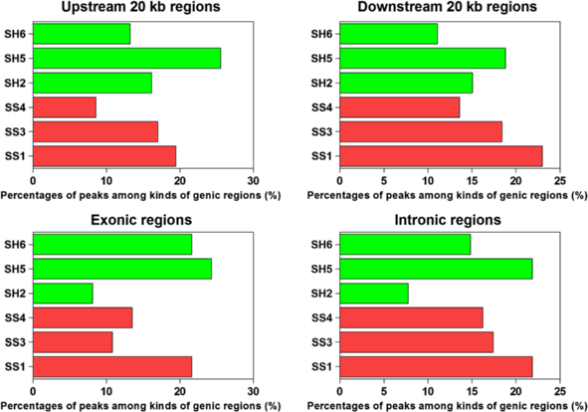
Peak distribution among different genic regions (upstream 20 kb, downstream 20 kb, exonic and intronic regions)

We generated global maps of H3K27me3 modifications via the ChIP-seq approach in *S. aureus* mastitis and healthy cows to reveal the regulation patterns of H3K27me3 related to *S. aureus* mastitis resistance. Around 7.6 million short reads from each sample were uniquely mapped to the bovine reference genome (Btau4.0), and these unique reads were used for further analysis (Additional file 1: Table S4). In order to analyze the distribution of H3K27me3 genome-wide, the bovine genome was divided into five kinds of regions – up 20 kb (20 kb upstream of TSS), exon, intron, down 20 kb (20 kb downstream of transcription end site (TES)) and intergenic regions – on the basis of annotation of “known genes” from the ENSEMBL Btau4.0 database. As shown in Fig. 4, five regions with the greatest abundance of reads were, in order, up 20 kb, intergenic, down 20 kb, intronic and exonic regions for both groups, which implied H3K27me3 might play key roles in the up- and downstream regions of genes to depress gene expression. To identify the real enriched regions of H3K27me3 (“H3K27me3 region”), the MACS model-based algorithm was utilized for identification of H3K27me3 peaks with significantly enriched ChIP signals. About 800 peaks were identified, and the total length of peaks was around 300 kb. The average length of each peak was around 400 bp. For each peak, the genes related to this peak were acquired and the functional regions overlapping with this peak were also captured. Consequently, around 300 genes were found that were associated with H3K27me3 peaks for each individual, and most peaks were enriched in the up 20 kb and down 20 kb regions (in total, 45 and 46 % in *S. aureus* mastitis and healthy cows, respectively). The peak enrichment was least in the exonic regions (less than 15 % in both groups) (Fig. 5). In conclusion, H3K27me3 modification was mainly found in the upstream and downstream regions of the target genes. And H3K27me3 might tend to regulate genes transcriptions via regulation regions of genes in bovine lymphocytes.

### Differences of H3K27me3 target genes between *S. aureus* mastitis and healthy dairy cattle

**Fig. 6:**
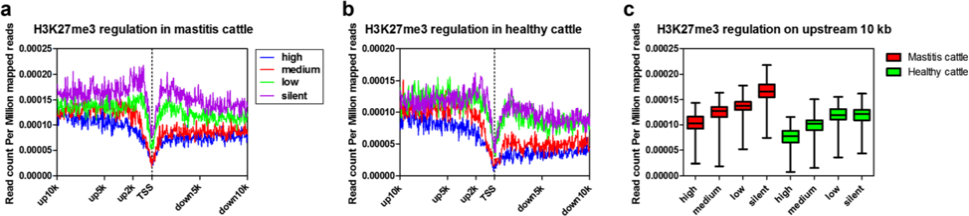
Modifications of H3K27me3 near transcription start sites (TSSs). Profiles of the H3K27me3 indicated across the TSS for highly active, two stages of intermediately active (medium and low) and silent gene sets were shown for **a** *S. aureus* mastitis group and **b** healthy group of cattle. Each gene set includes 700 genes according to their expression levels in primary lymphocytes of cow peripheral blood. Here, 20-kb regions of 700 genes in each group were aligned relative to their TSSs (*x* axis). The *y* axis shows the detected read count per million mapped reads. **c** The box plots described the modifications of H3K27me3 on upstream 10 kb regions for each gene set for two groups of cattle

It is known that gene promoters near the transcriptional start site (TSS) contain critical regulatory elements necessary for transcription. To document the functional consequences of H3K27me3 of target genes between *S. aureus* mastitis and healthy cows, H3K27me3 data and gene expression data were combined for analysis. To analyze the correlation between H3K27me3 modification and gene transcription, four sets of genes with different expression levels were chosen randomly (see Methods). H3K27me3 tag numbers in each gene region were counted and normalized near the TSS for these four sets of genes corresponding to highly expressed, two types of intermediately expressed (medium and low) and silent genes in the two groups of cattle (Fig. 6). As expected, H3K27me3 signals were negatively correlated with gene expression (Fig. 6a-b), which was in agreement with previous studies in bovine and in human T cells [21, 26]. Moreover, there were significantly enriched peaks around the TSS throughout the four gene sets in the two groups, which suggested that proximal regions to TSS were vital target points of H3K27me3 in bovine lymphocytes.

It is notable that H3K27 trimethylation levels in silent genes were comparatively higher in *S. aureus* mastitis cattle than in healthy cows. The distinct stratifications of methylation levels among the four sets of genes in *S. aureus* mastitis cattle were higher compared to in healthy cattle, which suggested that H3K27me3 regulation in mastitis cows infected by *S. aureus* might be more active than that in healthy cows. This showed a strong correlation between H3K27me3 and silent genes in *S. aureus* mastitis cows (Fig. 6a–b).

Further, up 10 kb regions relative to TSS were extracted and methylation features were compared between the two groups of cattle (Fig. 6c). The results showed that *S. aureus* mastitis individuals have higher H3K27me3 levels than healthy individuals for every gene set, which suggested that down-regulated and silent genes regulated by H3K27me3 may be related to the developmental process of *S. aureus* mastitis.

### Key regions of H3K27me3 target genes related to bovine *S. aureus* mastitis

**Fig. 7:**
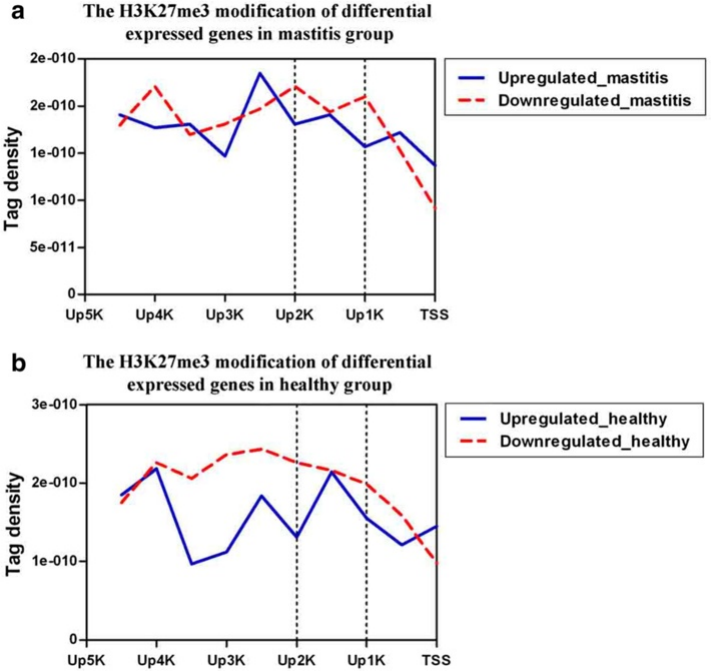
H3K27me3 modification profiles of the differentially expressed genes in subclinical *S. aureus* mastitis and healthy cows. **a** H3K27me3 modification of the differentially expressed genes for mastitis cows. Profiles of the H3K27me3 covered the region of up 5 kb to TSS were shown for 25 up-regulated and 36 down-regulated genes. For H3K27me3 modification for each gene, the tag density (number of tags per base pair) was calculated in 500 bp windows in upstream 5 kb regions. **b** H3K27me3 modification of the differentially expressed genes for healthy cows

To clearly reveal the relationship between H3K27me3 modifications and *S. aureus* mastitis, we calculated H3K27me3 modification levels in 61 differentially expressed genes between mastitis and healthy cows. As shown in Fig. 7, the H3K27me3 modification levels in the down-regulated genes were significantly higher than those in the up-regulated genes either for the mastitis or healthy cows. However, the remarkable modification region was only located in the up 2 kb region relative to TSS. Thus, these differentially expressed genes regulated by H3K27me3 in the up 2 kb regions may be related to *S. aureus* mastitis susceptibility and are key candidate genes for future anti-mastitis study.

### Validation of ChIP-seq and DGE results

**Fig. 8:**
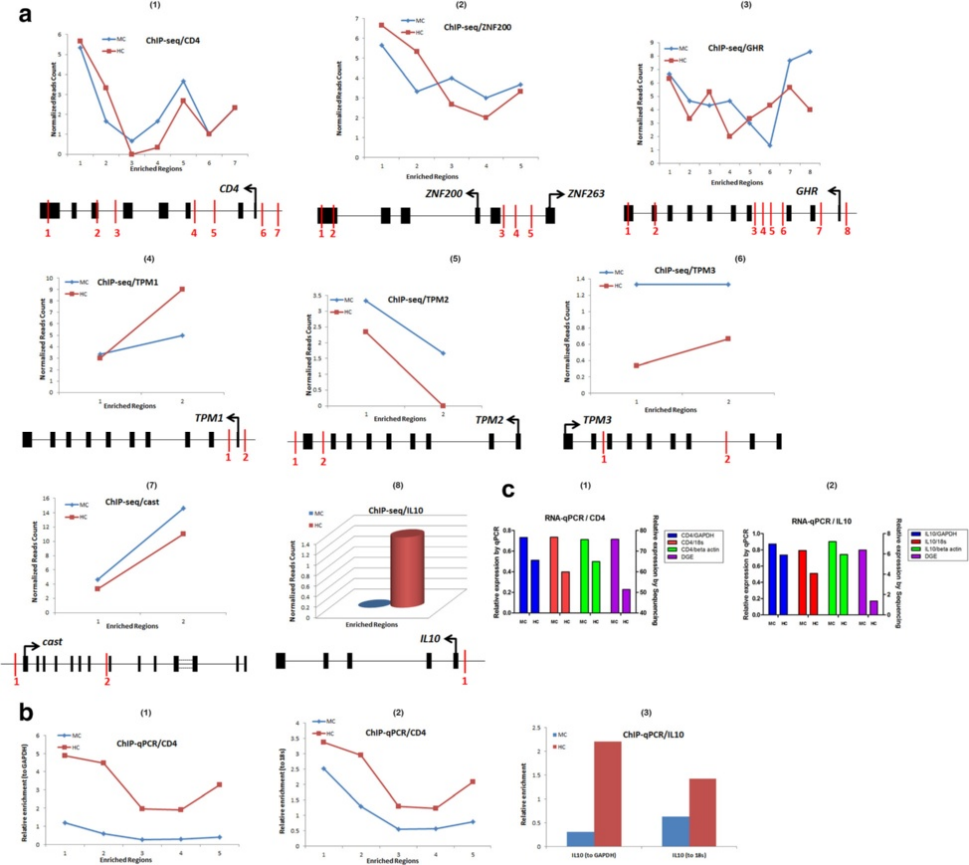
H3K27me3 levels on several differentially expressed genes by ChIP-seq and their qPCR verification. **a** The H3K27me3 modification profiles were shown on some enriched regions of *CD4, ZNF200, GHR, TPM1, TPM2, TPM3, cast* and *IL10* genes. The structures of genes are displayed below the H3K27me3 profiles, in which black boxes represent exons and arrows represent the transcriptional direction of genes. The red vertical bars represent selected enriched regions. **b** Real-time qPCR data showed H3K27me3 enrichment on selected enriched regions of *CD4* and *IL10* genes for two groups of cattle. Five sites of *CD4* validated by real-time PCR are corresponding to sites 1–5 of ChIP-seq in Fig. 8a1, while one site of *IL10* validated by qPCR is same as that in ChIP-seq of Fig. 8a8. Negative controls used were *GAPDH_P1* and *18 s rRNA_P1*. **c** Real-time RT-qPCR were performed in two groups of cattle for validation of *CD4* and *IL10* expression (*GAPDH*, *18 s rRNA* and *beta-actin* were used as housekeeping genes). The individual with the lowest corrected Ct value was selected as a control sample to calculate relative expression of genes. DGE indicates the results of digital gene expression of *CD4* and *IL10* (for TPM)

To assess the accuracy of the ChIP-seq analysis results and validate differential H3K27me3 modifications and gene expression levels between *S. aureus* mastitis and healthy cows, bovine *CD4* and *IL10* cytokine genes were used to confirm the H3K27me3 enrichment profiles using ChIP-quantitative PCR (ChIP-qPCR) approach and their mRNA levels by RT-qPCR. Six enriched regions from the H3K27me3 maps for the two genes (5 sites at the gene body region of *CD4* and 1 site at the promoter region of *IL10*) were chosen based on different H3K27me3 enrichment levels among different regions and between the two groups of cattle. The primer pairs we used in the validation are listed in Additional file 1: Table S5. Relative enrichment was quantified for each site with real-time PCR reactions and normalized by the negative controls (*GAPDH*_P1 and *18 s*_P1). For the five sites in *CD4* gene (Fig. 8a1, b1 & b2), the relative enrichments were mostly consistent with the profiles observed in ChIP-seq in that the enrichments at P1, P2 and P5 were higher while lower at P3 and P4. For one site on promoter region of *IL10* gene (TSS: 3572215), the relative enrichment levels were consistent with the results in ChIP-seq that the enrichments for healthy cattle were higher than for mastitis cattle (Fig. 8a8 & b3). In order to confirm the results of gene expression by sequencing and validate the relationship between H3K27me3 enrichment and gene expression, the mRNA levels of *CD4* and *IL10* genes were also measured with reverse transcription-quantitative PCR (RT-qPCR) and standardized with three housekeeping genes (*GAPDH*, *18 s rRNA* and *beta actin*). RT-qPCR results showed that the expression of *CD4* and *IL10* were mostly consistent with the data in the DGE (Fig. 8c) and were negatively related with the level of corresponding H3K27me3. Consequently, H3K27me3 levels in healthy cows were higher than in *S. aureus* mastitis cows for both genes (*CD4* and *IL10*), which means that increased H3K27me3 level repressed pro-inflammatory gene expression (*IL10*) in healthy dairy cattle and vice versa in *S. aureus* mastitis cows.

## Discussion

*S. aureus* is a pathogen that not only affects dairy cattle but also presents a serious threat for human health. H3K27me3, a representatively repressive epigenetic mark [27], could be of critical significance in understanding the interactions between bovine innate or adaptive immunity and anti *S. aureus* infection. The present study reports the first specific H3K27me3 modification profiles and their target gene expression in bovine lymphocytes between *S. aureus* subclinical mastitis and healthy lactating cows.

With regard to subclinical mastitis cows, it is widely accepted that the somatic cell count (SCC) in milk is a useful indicator for dairy herds. General agreement relies on the values of SCCs that are less than 200,000 cells/mL for healthy cows. SCC greater than 500,000/ml indicates a problem with subclinical or clinical mastitis and inferior milk quality [28]. Indeed, we chose candidate cows for *S. aureus* mastitis infected and non-infected cows based on SCC information. However, we found that the SCC of 60 % of all *S. aureus* mastitis cases was higher than 200,000 of cells/ml. Our data indicated that the identification of subclinical mastitis cows induced by *S. aureus* was not very accurate based only on SCC indicator.

To clarify the standards for *S. aureus* subclinical mastitis, we studied DHI records of the samples in our study (Additional file 1: Table S6). Our results showed that the SCCs of SS1, SS3 and SS4 were persistently higher than 300,000 cells/ml during the previous three months, in which the SCCs of SS4 sample dropped to 220,000/ml on the sampling day. However, the milk samples of the three cows were identified as infected by *S. aureus*, suggested that they were in *S. aureus* mastitis condition. The decreased SCC of SS4 may be associated with immunity to *S. aureus* in her udder. Furthermore, the SCCs of SH2, SH5 and SH6 were persistently lower than 100,000/ml during at least three months, in which the SCC of SH2 sample rose to 480,000/ml on the sampling day. Considering the milk samples of SH2, SH5 and SH6 were not found to contain *S. aureus*, the high SCC of SH2 may be related to another organism or stress during sampling. These results indicated that the culture of bacteria was more accurate than SCC for determination of *S. aureus* subclinical mastitis. To accurately detect *S. aureus* mastitis cows, we propose that cows with persistently raised SCCs (three consecutive monthly counts >300,000 cells/ml [29] can be candidate *S. aureus* mastitis cows.

Taking advantage of digital gene expression profiling techniques, we screened differentially expressed genes and conducted cluster analysis of these genes between subclinical mastitis and healthy cows based on the classifications of SCC and *S. aureus*, respectively. A total of 55 differentially expressed genes were found by SCC classification, while 61 differentially expressed genes were screened based on *S. aureus* classification. GO analysis of the differentially expressed genes based on SCC classification demonstrated that the up-regulated genes were mainly enriched in molecular function and participated in protein-DNA complex and some important immune processes, and the down-regulated genes were enriched in biological process and involved in cellular macromolecular localization and cellular component biogenesis. According to the classification with or without *S. aureus* infection, GO analysis showed that the up-regulated genes were involved in some immune processes, such as *KLRK1*, *CFB*, *IL10*, *PTX3* and *ARG1*, while the down-regulated genes were related to redox enzyme activity, transmembrane transporter activity, transcription regulator and development and reproduction. Together with the results of two classifications, 13 differentially expressed genes were shared (Additional file 1: Table S7). Among these genes, *CFB* participated in positive regulation of immune response and complement activity; *ARG1* was involved in cell response to organonitrogen and bacterial source molecular; *PTX3* was related to positive regulation of endocytosis and inducing programmed cell death; *PPARD* was observed in transcriptional regulation and cell adhesion; *CST3* was correlated with protease inhibitor and DNA replication and repair. These five genes involved in immune and transcription processes could be significant candidate genes for studying bovine subclinical mastitis. These results suggested that differentially expressed genes screened by *S. aureus* classification were more associated with immune traits and immune disease.

We hypothesized that the down-regulated genes in the subclinical *S. aureus* mastitis cows might be suppressed by H3K27me3 modification. Therefore, the H3K27me3 levels of differentially genes between *S. aureus* mastitis and healthy cow were compared. As expected, the down-regulated genes were negatively correlated with the H3K27me3 levels, which was consistent with previous observations [21]. Among these genes, *TPM* family is correlated with muscle contraction and an important regulatory protein. Up to date, four TM genes have been verified in mammals, which were named as *TPM1*, *TPM2*, *TPM3* and *TPM4*. The mutant *TPM1* gene can generate famialial hypertrophic cardiomyopathy; *TPM2* is associated with hypertensive cardiomyopathy and arthrogryposis; *TPM3* is related to nemaline myopathy and skeletal muscle weakness [30, 31]. Our ChIP-seq results of H3K27me3 indicated that H3K27 methylation levels have significant difference on candidate-enriched regions of *TPM* genes between *S. aureus* subclinical mastitis and healthy dairy cows (Fig. 8a4–6). Thus, *TPM* family genes might be candidate genes associated with bovine subclinical mastitis, which is worth further study.

In the present study, some unique H3K27me3 genes for subclinical *S. aureus* mastitis cattle or healthy cattle were also documented. H3K27me3 modifications in most of these genes appeared to be higher in *S. aureus* mastitis individuals only on promoter regions, such as *ZNF200*, *GHR*, *TPM* genes and *cast* gene. However, for *CD4* and *IL10* genes, methylation levels on promoter regions were higher in healthy individuals (Fig. 8a1–8 & 8b), which implied that H3K27me3 would depend on different conditions to exert its roles and it might tend to repress the expression of some pro-inflammatory genes in healthy conditions.

Combined with KEGG pathway analysis, we conducted the network analysis for the key candidate genes discovered by DGE and ChIP-seq data. The results indicated that these candidate genes were involved in signaling of MHC pathway, T cell receptor pathway and cytokines-cytokine receptor interaction signaling pathway, in which γ-interferon mediates MHC I to act on CD8 T cells and nature killer cells for killing the target cells. In addition, other cytokine genes such as *IL10*, *IL13RA1* and *CXCL10* participate in the synthesis of γ-interferon. *ARG1* gene is involved in cell responses to the identification of bacterial source and then induces the immune interaction. More importantly, H3K27me3 regulated expression of these key genes involved in these important immune pathways.

**Fig. 9:**
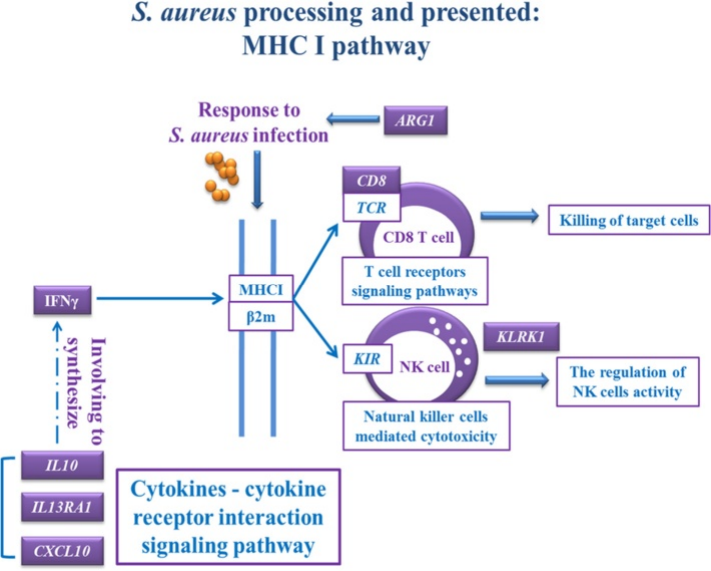
Hypothesis of “The regulation of H3K27me3 on *S. aureus* subclinical mastitis susceptibility in dairy cows lymphocytes”. This cartoon shows the interaction of signaling networks induced by *S. aureus* infection including MHC I pathway, T cell receptor pathway and cytokines-cytokine receptor interaction signaling pathway, in which γ-interferon mediates MHC I to act on CD8 T cells and nature killer cells for killing the target cells. Cytokine genes *IL10*, *IL13RA1* and *CXCL10* participate in the synthesis of γ-interferon. *ARG1* gene is involved in cell responses to identify *S. aureus* and then induces the immune interaction. However, H3K27me3 regulates expressions of these key genes in this immune signaling network

According to the biological pathways of differentially expressed genes and regulation of H3K27me3, we propose the hypothesis of “The regulation of H3K27me3 on subclinical *S. aureus* mastitis susceptibility in dairy lymphocytes” (Fig. 9), which provides the basic information for the epigenetic study on bovine *S. aureus* mastitis susceptibility. Further studies are warranted to confirm and better understand the regulation mechanism of H3K27me3 on *S. aureus* mastitis in mammary gland cells of dairy cattle.

## Conclusions

Our data provide a novel insight into the "cross-talk" between *S. aureus* and lymphocytes in dairy cattles based on histone H3 methylation regulation. The differentially expressed genes (such as IL10 and PTX3) in lymphocytes regulated by H3K27me3 may relate to *S. aureus* mastitis susceptibility. These epigenetic targted genes could be considered as potential biomarkers used in anti-*S. aureus* mastitis breeding.

## Methods

### Sampling

Seventeen Holstein cows were selected from a dairy herd in Beijing (China) based on their DHI records through the whole year. Performance testing data (DHI) was offered by the official Dairy Data Center of China (Beijing, China) including daily milk yields, fat percentage, protein percentage and somatic cell counts (SCC). They were fed on the same lactation diet according to energy recommendations for lactating Chinese Holstein cows and were handled in accordance with the guidelines of the Animal Care and User Committee. 100 mL of fresh milk used for SCC measure and bacteria identification was aseptically collected from all lactating quarters and mixed [32], simultaneously, 40 mL of blood sample was obtained from the jugular vein for each animal with EDTA anticoagulant tubes following the regular quarantine inspection of the farm, so no ethical approval was required for this study. Bacteriological culture of milk samples was performed according to National Mastitis Council standards [33]. A volume of 100 μL of milk was streaked onto a plate of blood agar, and the number of colony-forming units of each of the bacterial species was counted at 18 and 24 h for 37 °C. The udder was considered to be not infected by bacteria and the cow was considered mastitis resistant when ≤ 500 CFU of colonies/mL was detected in the milk. Milk samples containing more than two bacterial species were considered to be contaminated [34]. Suspect colonies of *S. aureus* were purified, and cultured at 37 °C for an additional 18–24 h and were identified by positive Gram stain, and isolates of *S. aureus* were finally confirmed by a positive catalase test. In order to double check the *S. aureus* in milk, the molecular methods were developed for simultaneous bacterial species identification and detection. A modified protocol was applied for bacterial DNA isolation from milk samples. PCR assay was performed for amplifying *nuc* gene and 16S rRNA gene sequencing method was additionally applied to identify simultaneous bacterial species [35].

### ChIP-seq and DGE-seq

Peripheral blood lymphocytes were isolated by Lymphocytes Separation Medium (TBDsciences, Tianjin, China, PN.LTS1086) according to the manufacturer’s instructions, and the purity was 90–95 %. The protocols of ChIP-seq and DGE-seq were the same as previously described [21]. All sequencing data are available from GEO repository (accession number GSE71341).

### The bioinformatics analysis

For ChIP-seq experiment, H3K27me3 peaks were identified by MACS1.4.0 with a *bandwidth* of 200 bp, *mfold* of 30 and *p-value cutoff* of 1.00e-05 based on mapped files (Btau4.0), meanwhile, wiggle files for each chromosome were output at every 50 bps for viewing the enrichment of H3K27me3 in the UCSC genome browser or IGV with *--wig* parameter. In addition, genes related to peaks were also found for further analysis. Raw data from DGE-seq were mapped to the bovine reference genome by SOAP 2.21 software and then they were annotated via Ensembl BioMart database. The expression level of one gene was represented by TPM (number of transcript copies in per million clean tags) [36, 37]. All known genes were divided into multiple sets according to their expression levels, in which four sets containing 700 genes in each gene set were picked corresponding to high (TPM: 70–3000), medium (TPM: 7–12), and low expressed sets (TPM: 0.5–1.5) and silent gene set for the combination analysis of H3K27me3 modification and gene expression. H3K27me3 tags detected were aligned in each gene set across transcription start sites (TSS) or gene bodies. To reveal the relationship between H3K27me3 modification and mastitis resistance, we calculated H3K27me3 modification levels in 61 differentially expressed genes between mastitis and healthy cows by *S. aureus* classification. For mastitis group, 25 up-regulated genes and 36 down-regulated genes were applied to calculate H3K27me3 densities, while there were 25 down-regulated and 36 up-regulated genes in healthy group. Profiles of the H3K27me3 were plotted in each of 500 bp windows covering the region from 5 kb upstream to the TSS of the genes.

### Real-time PCR validation

Bovine *CD4* and *IL10* cytokine genes were used to confirm their H3K27me3 enrichment profile using ChIP-qPCR approach and their mRNA levels by RT-qPCR. Q-PCR reactions were carried out using Roche LightCycler 480 qPCR machine with SYBR green dye. Three duplicates for each site were used in ChIP-qPCR and RT-qPCR reactions. A negative primer pair (*GAPDH*_P1 or 18 s *rRNA*_P1) was applied to determine the relative fold enrichments for ChIP-qPCR. The mRNA expression of *CD4* or *IL10* was normalized against three housekeeping genes (*GAPDH*, *18 s rRNA* and *beta actin*) cDNA in the corresponding samples. The detailed programs were the same as previously described.

## Additional file

Additional file 1:
**Table S1.** Primers used to identify *S. aureus*. **Table S2.** Data statistics of DGE sequencing. **Table S3.** KEGG pathway analysis of differential expressed genes related to *S. aureus* mastitis resistance. **Table S4.** Raw data and mapping results of bovine H3K27me3. **Table S5.** Primers used to confirm ChIP-seq and DGE results of *CD4* and *IL10* genes. **Table S6.** DHI records and bacterium culture of sample cows. **Table S7.** Common differentially expressed genes by the two classification criteria. (DOCX 38 kb)

## Abbreviations

ChIP-Seq: Chromatin immunoprecipitations coupled with sequencing
DGE: Digital gene expression
H3K27me3: Trimethylaiton of lysine 27 on histone H3
*S. aureus*: *Staphylococcus aureus*
SCC: Somatic cell count
TES: Transcription end site
TSS: Transcription start site
