# Supplementary material for: Whole-genome regulation analysis of histone H3 lysin 27 trimethylation in subclinical mastitis cows infected by Staphylococcus aureus
Source: BMC Genomics. 2016 Aug 8;17:565. doi: 10.1186/s12864-016-2947-0 (PMC4977872; doi:10.1186/s12864-016-2947-0)
Supplement: Additional file 1: — Table S1. Primers used to identify S. aureus. Table S2. Data statistics of DGE sequencing. Table S3. KEGG pathway analysis of differential expressed genes related to S. aureus mastitis resistance. Table S4. Raw data and mapping results of bovine H3K27me3. Table S5. Primers used to confirm ChIP-seq and DGE results of CD4 and IL10 genes. Table S6. DHI records and bacterium culture of sample cows. Table S7. Common differentially expressed genes by the two classification criteria. (DOCX 38 kb) [file 12864_2016_2947_MOESM1_ESM.docx]

**Table S1** Primers used to identify *S. aureus*

| Gene | Primer name | Sequence (5’→3’) | The length of PCR product (bp) | Annealing temperature (℃) | Represent strain |
| --- | --- | --- | --- | --- | --- |
| *NUC* | NUC_F  NUC_R | 5'-ATATGTATGGCAATCGTTTCAAT-3'  5'-GTAAATGCACTTGCTTCAGGAC-3' | 279 | 55 | *S. aureus* |
| *MecA* | MecA_F  MecA_R | 5’-GTAGAAATGACTGAACGTCCGATAA-3’  5’-CCAATTCCACATTGTTTCGGTCTAA-3’ | 310 | 55 | Methicillin-resistant *S. aureus* (MRSA) |
| *CFB* | CFB_F  CFB_R | 5'-GCTGGTGCATTGTTATTTTCA-3'  5'-ATGGTAGCTCTATCAGTTGGTTTT-3' | 512 | 55 | Streptococcus agalactiae |
| *Universal_16s* | Bacter_F  Bacter_R | 5'-GAGTTTGATCMTGGCTCAG-3'  5'-CTAHAGGGTATCTAATCCT-3' |  | 55 |  |

Note: The sequence of *nuc* gene is highly conserved DNA fragments of *S. aureus*, which encodes TNase enzyme of *S. aureus* specific.

*S. aureus* of methicillin-resistant is known as MRSA. There is the relationship between MecA gene and MRSA, therefore, the method that identifying MRSA strains by *MecA* gene is not impacted by the susceptibility testing conditions, and it has high specificity, Thus, the test of *MecA* gene is consider as the "gold standard" of judgment MRSA.

*cfb* gene is known as bovine source Streptococcus agalactiae CAMP factor.

**Table S2** Data statistics of DGE sequencing

|  | SS1 | SH2 | SS3 | SS4 | SH5 | SH6 |
| --- | --- | --- | --- | --- | --- | --- |
| Total tags | 3664274 | 3520031 | 3602312 | 3517383 | 3551552 | 3635830 |
| Clean tags | 3505104 | 3346804 | 3460445 | 3367436 | 3413072 | 3484189 |
| Mapped tags | 2281109 (65.08%)^*^ | 2311983 (69.08%) | 2390782 (69.09%) | 2268661 (67.37%) | 2302501 (67.46%) | 2374927 (68.16%) |
| Unique mapped tags | 1117051 (31.87%) ^&^ | 1092242 (32.64%) | 1088671 (31.46%) | 1053905 (31.30%) | 1141492 (33.44%) | 1158862 (33.26%) |
| Genes mapped by unique tags | 6862 (37.17%)^§^ | 6894 (37.35%) | 6943 (37.61%) | 7007 (37.96%) | 6764 (36.64%) | 6923 (37.50%) |

^*^Mapping rate: the ratio of mapped tags to clean tags

^&^Unique mapping rate: the ratio of unique mapped tags to clean tags

^§^Valid genes rate: the ratio of genes mapped by unique tags to the reference genes number (18460)

**Table S3** KEGG pathway analysis of differential expressed genes related to *S. aureus* mastitis resistance

| No. | Pathways involoved by up-regulated genes | Pathways involoved by down-regulated genes |
| --- | --- | --- |
| 1 | Allograft rejection | Arachidonic acid metabolism, |
| 2 | Arginine and proline metabolism | Basal transcription factors, |
| 3 | Asthma | Calcium signaling pathway, Neuroactive ligand-receptor interaction, |
| 4 | Autoimmune thyroid disease | Cell cycle, |
| 5 | Cardiac muscle contraction | Cytokine-cytokine receptor interaction, |
| 6 | Complement and coagulation cascades | Endocytosis, |
| 7 | Cytokine-cytokine receptor interaction | Fatty acid metabolism |
| 8 | Dilated cardiomyopathy | Glutathione metabolism, Metabolism of xenobiotics by cytochrome P450, Drug metabolism, |
| 9 | Fructose and mannose metabolism | Jak-STAT signaling pathway |
| 10 | Hypertrophic cardiomyopathy (HCM) | MAPK signaling pathway, ErbB signaling pathway |
| 11 | Intestinal immune network for IgA production | Neuroactive ligand-receptor interaction, Vascular smooth muscle contraction, |
| 12 | Jak-STAT signaling pathway | Oxidative phosphorylation, |
| 13 | Natural killer cell mediated cytotoxicity | Pathways in cancer, Colorectal cancer, Endometrial cancer, Thyroid cancer, Bladder cancer |
| 14 | Neuroactive ligand-receptor interaction | PPAR signaling pathway, Wnt signaling pathway, Pathways in cancer, Acute myeloid leukemia, |
| 15 | Purine metabolism, Pyrimidine metabolism, Lysosome | Primary bile acid biosynthesis, PPAR signaling pathway, |
| 16 | Riboflavin metabolism | Regulation of actin cytoskeleton, |
| 17 | RNA degradation | Systemic lupus erythematosus, |
| 18 | Systemic lupus erythematosus | Tryptophan metabolism, Methane metabolism, Amyotrophic lateral sclerosis (ALS), |
| 19 | T cell receptor signaling pathway | Wnt signaling pathway, TGF-beta signaling pathway |
| 20 | Thiamine metabolism |  |

**Table S4** Raw data and mapping results of bovine H3K27me3

| Sample ID | Clean reads | Mapped reads | Mapping rate^&^ | Unique mapped reads | Unique mapping rate^§^ |
| --- | --- | --- | --- | --- | --- |
| SS1 | 9738458 | 9360058 | 96.11% | 7633622 | 78.39% |
| SH2 | 9746553 | 9371529 | 96.15% | 7589192 | 77.87% |
| SS3 | 9747187 | 9367267 | 96.10% | 7703707 | 79.04% |
| SS4 | 9739361 | 9353500 | 96.04% | 7418515 | 76.17% |
| SH5 | 9744633 | 9381894 | 96.28% | 7627477 | 78.27% |
| SH6 | 9739000 | 9369515 | 96.21% | 7538888 | 77.41% |

^&^Mapping rate: the ratio of mapped reads to clean reads.

^§^Unique mapping rate: the ratio of unique mapped reads to clean reads.

**Table S5** Primers used to confirm ChIP-seq and DGE results of *CD4* and *IL10* genes.

| qPCR | Primer name | Sequence (5’→3’) | The length of PCR product (bp) | The template of PCR | Annealing temperature (℃) |
| --- | --- | --- | --- | --- | --- |
| *CD4*_ChIP | CD4_P1 | F：5' TGG ACT AGC CCA GGT CTC TT 3'  R：5' GCA GGT TTT GTT CCC TGG TA 3' | 249 | DNA | 60° |
| *CD4*_ChIP | CD4_P2 | F: 5' GAA GCT GTG CTT CCT CCA TT 3'  R: 5' CCA TCT TCA AGT TCA GGG TCA 3' | 223 | DNA | 60° |
| *CD4*_ChIP | CD4_P3 | F: 5' ACT GCA AGG AGA CCC AAC C 3'  R: 5' CCA GCC ATC TCA TCC TCT GT 3' | 193 | DNA | 60° |
| *CD4*_ChIP | CD4_P4 | F: 5' GGA GAA TCC CAT GGA CAG AA 3'  R: 5' TCT GCC CTG GTC CTA ATC TG 3' | 221 | DNA | 60° |
| *CD4*_ChIP | CD4_P5 | F: 5' TCC CTT CCC AAC TTC CTG AT 3'  R: 5' CCA GGA AGC TCC CAC TGT AA 3' | 239 | DNA | 60° |
| *IL10*_ChIP | IL10_P1 | F: 5' TTC CCA AAA TGT GCA TAC CTC 3'  R: 5' CTT AGC AGC AGC AGC CAA TC 3' | 248 | DNA | 60° |
| *GAPDH*_ChIP | GAPDH_P1 | F: 5' TAC CGA AGA GCC TCG AGA A 3'  R: 5' GCC GCA AGG ATA TAA CAG GA 3' | 155 | DNA | 60° |
| *18s rRNA*_ChIP | 18s rRNA_P1 | F: 5' ACG TAA CTT AAG CTT CCT CC 3'  R: 5' CGA TTC TAG CAG TTT GGA TT 3' | 125 | DNA | 60° |
| *CD4*_mRNA^*^ | CD4_m | F：5' ACT GAG CCA TCG AGT GGA AT 3'  R：5' CAG TGA CAG GCT CTT GAC GT 3' | 289 | cDNA | 60° |
| *IL10*_mRNA^*^ | IL10_m | F: 5' TGT TGA CCC AGT CTC TGC TG 3'  R: 5' AGC TTC TCC CCC AGT GAG TT 3' | 154 | cDNA | 60° |
| *GAPDH*_mRNA^*^ | GAPDH_m | F: 5’ GGCGTGAACCACGAGAAGTATAA 3’  R: 5’ CCCTCCACGATGCCAAAGT 3’ | 119 | cDNA | 60° |
| *18s rRNA*_mRNA^*^ | 18s rRNA_m | F: 5’ GTAACCCGTTGAACCCCATT 3’  R: 5’ CCATCCAATCGGTAGTAGCG 3’ | 152 | cDNA | 60° |
| *Beta-actin*_mRNA^*^ | ACTB_m | F: 5’ AGCAAGCAGGAGTACGATGAGT 3’  R: 5’ ATCCAACCGACTGCTGTCA 3’ | 239 | cDNA | 60° |

* Used for verifying gene expression levels, and the templates used were cDNA for real-time PCR.

**Table S6** DHI records and bacterium culture of sample cows

| ID | Milk days | Parity | Milk yield per day (Kg) | SCC (×1000/ml) | Test date | The culture of *S. aureus* |
| --- | --- | --- | --- | --- | --- | --- |
| SS1 | 66 | 3 | 44 | 2281 | 2010-5-10 | Positive |
|  | 129 | 3 | 29 | 1707 | 2010-7-12 |  |
|  | 157 | 3 | 28.5 | 1249 | 2010-8-9 |  |
|  | 185 | 3 | 15 | 1790 | 2010-9-6 |  |
|  | 220 | 3 | 24 | 1755 | 2010-10-11 |  |
|  | 305 | 3 | 28.1 | 436 | 2010-12-3 |  |
| SH2 | 23 | 3 | 42 | 51 | 2010-8-9 | Negative |
|  | 51 | 3 | 45 | 47 | 2010-9-6 |  |
|  | 86 | 3 | 41 | 46 | 2010-10-11 |  |
|  | 166 | 3 | 42.7 | 482 | 2010-12-9 |  |
| SS3 | 107 | 1 | 36 | 2824 | 2010-7-12 |  |
|  | 163 | 1 | 29 | 1018 | 2010-9-6 |  |
|  | 198 | 1 | 33 | 807 | 2010-10-11 |  |
|  | 283 | 1 | 32.7 | 1106 | 2010-12-3 | Positive |
| SS4 | 75 | 1 | 33 | 2210 | 2010-6-8 | Positive |
|  | 137 | 1 | 32.5 | 1201 | 2010-8-9 |  |
|  | 200 | 1 | 40 | 800 | 2010-10-11 |  |
|  | 285 | 1 | 35.2 | 224 | 2010-12-3 |  |
| SH5 | 18 | 1 | 24 | 65 | 2010-9-6 | Negative |
|  | 53 | 1 | 30 | 45 | 2010-10-11 |  |
|  | 81 | 1 | 34 | 25 | 2010-11-8 |  |
|  | 133 | 1 | 29.3 | 27 | 2010-12-9 |  |
| SH6 | 35 | 1 | 43 | 37 | 2010-10-11 | Negative |
|  | 63 | 1 | 44 | 29 | 2010-11-8 |  |
|  | 102 | 1 | 43.5 | 104 | 2010-12-3 |  |

**Table S7** Common differentially expressed genes by the two classification criteria

| No. | Ensembl_Gene_ID | WikiGene_name | WikiGene_description |
| --- | --- | --- | --- |
| 1 | ENSBTAG00000019906 | *VPS25* | vacuolar protein sorting 25 homolog (S. cerevisiae) |
| 2 | ENSBTAG00000012403 | *ARG1* | arginase, liver |
| 3 | ENSBTAG00000007450 | *CFB* | complement factor B |
| 4 | ENSBTAG00000008966 | *TSPAN7* | tetraspanin 7 |
| 5 | ENSBTAG00000009012 | *PTX3* | pentraxin 3, long |
| 6 | ENSBTAG00000032089 | *CDC42EP2* | CDC42 effector protein (Rho GTPase binding) 2 |
| 7 | ENSBTAG00000017542 | *PPARD* | peroxisome proliferator-activated receptor delta |
| 8 | ENSBTAG00000044100 | *HEATR1* | hypothetical LOC508697 |
| 9 | ENSBTAG00000031160 | *BC142384* | Immunoglobulin light chain, lambda gene cluster |
| 10 | ENSBTAG00000013366 | *NRIP3* | nuclear receptor interacting protein 3 |
| 11 | ENSBTAG00000038375 | *STOM* | stomatin |
| 12 | ENSBTAG00000039953 | *GBP1* | Guanylate binding protein 1, interferon-inducible |
| 13 | ENSBTAG00000000598 | *CST3* | cystatin C |
